# Diverging Trajectories of Depressive Symptoms During Electroconvulsive Therapy: Toward Personalized Treatment

**DOI:** 10.1016/j.bpsgos.2026.100722

**Published:** 2026-03-13

**Authors:** Kjersti Sellevåg, Christoffer Andreas Bartz-Johannessen, Ketil Joachim Oedegaard, Axel Nordenskjöld, Leif Oltedal, Jeanette Solheimslid Bjørke, Ute Kessler

**Affiliations:** a Division of Psychiatry, Haukeland University Hospital, Bergen, Norway; b Department of Clinical Medicine, Faculty of Medicine, University of Bergen, Bergen, Norway; c Health Care Research Centre, Faculty of Medicine and Health, Örebro Universitet, Örebro, Sweden; d Mohn Medical Imaging and Visualization Centre, Department of Radiology, Haukeland University Hospital, Bergen, Norway; e Psychiatric Division, Stavanger University Hospital, Stavanger, Norway

**Keywords:** Depression, Electroconvulsive therapy, Neurostimulation treatment, Personalized treatment, Psychiatry, Treatment trajectories

## Abstract

**Background:**

While electroconvulsive therapy (ECT) is an effective treatment for depression, patient responses vary. In addition, there is a lack of guidance in making treatment decisions for patients with an initial nonresponsive trajectory. In this study, we aimed to identify distinct latent patient trajectories and explore outcomes among patients who had not responded after 9 treatments but who continued therapy.

**Methods:**

In this register-based cohort study, including 344 patients (61% female), we applied a latent class mixed model to identify latent patient trajectories and investigated variables associated with class affiliation in a multinomial regression analysis. We also identified nonresponders at treatment 9 and investigated final outcomes in a logistic regression model.

**Results:**

Three latent classes were identified, all of which showed improvement, although classes 2 and 3 showed faster and steeper symptom reduction, with mean Montgomery–Åsberg Depression Rating Scale (MADRS) scores post-ECT of 9.8 and 5.3, respectively. Class 1 showed a shallower response pattern, with a mean MADRS score post-ECT of 20.9; variables associated with affiliation to this class were longer duration of depressive episode, nonpsychotic depression, lower age, and lower baseline depression level. Of nonresponders to ECT at session 9, 44.9% achieved final response and 17.9% achieved remission; female sex and lower age groups (<60) were associated with higher odds of final nonresponse.

**Conclusions:**

Our study supports ECT as an effective treatment for depression, while highlighting the existence of distinct response trajectories. A significant proportion of patients with an initial nonresponse trajectory may still benefit from continued ECT. Our findings reinforce the importance of individualized treatment decisions guided by clinical response and close monitoring.

## Plain Language Summary

Electroconvulsive therapy (ECT) is an effective treatment for depression, although responses vary. Using a latent class mixed model, we identified 3 patient subgroups with different symptom trajectories during ECT. All improved, but class 1 showed slower, modest progress, associated with longer episode duration, nonpsychotic depression, younger age, and lower baseline depression severity. Among patients unresponsive at session 9, 44.9% later responded and 17.9% achieved remission. These findings confirm ECT’s efficacy while emphasizing variability in outcomes. Continued ECT may benefit patients who do not respond early. In summary, the findings underscore the need for individualized treatment decisions.

Electroconvulsive therapy (ECT) is considered the most effective acute treatment for severe or treatment-resistant unipolar and bipolar depression, its efficacy and effectiveness having been established through research including randomized trials (1,2) and registry studies (3, 4, 5). Various methods have been used to assess the effects of ECT on depressive symptoms, including examining dichotomous outcomes such as response or remission or group-level total scores (5, 6, 7). According to a narrative review by Espinoza and Kellner (8), ECT achieves response rates of 60% to 80% and remission rates of 50% to 60% in patients with treatment-resistant major depressive disorder. Traditional statistical approaches have identified clinical variables associated with ECT end-point outcomes such as response and remission, including better outcomes in older patients and patients with depression with psychotic features (7) and poorer outcomes in patients with longer episode duration (9). However, while informative at the group level, these methods may not fully capture the heterogeneity in individual clinical courses, as patients vary considerably in how they respond to ECT (5).

To further elucidate diverging symptom trajectories, a different angle is to apply statistical analyses that are suitable to reveal latent subclasses in patient populations, such as latent class mixed models (LCMMs) (10). In previous studies using similar statistical approaches in an ECT setting (11, 12, 13), distinct symptom trajectories and clinical associations were identified, reflecting clinical meaningful heterogeneity in ECT response. However, findings have been inconsistent, possibly due to variation in statistical approaches and methodological limitations such as small sample and class sizes and heterogeneity in ECT protocols. Research on such longitudinal ECT response trajectories remains scarce, and further research with larger patient samples has been called for (11). Identifying such subclasses and their characteristics can improve the understanding of heterogeneous response patterns and generate hypotheses for future predictive models, potentially informing more personalized treatment strategies as evidence evolves (14,15).

In responders, ECT is usually ceased upon remission—the ultimate goal of depression treatment (16). However, whether to continue or end ECT treatment for patients with an initial partial or nonresponse trajectory is an important clinical question—an issue for which there is currently no consensus or uniform guidelines (12). This is particularly challenging as clinicians must weigh the potential benefits of continuing ECT against risks such as cognitive side effects (17), cardiopulmonary events, and anesthesia-related complications associated with prolonged treatment (8). In a study by Kellner *et al.* (18), it was found that among remitted patients, more than 90% remitted before or at session 9. This time point also reflects a clinical decision point when reassessment of treatment is relevant, i.e., evaluating whether to maintain, intensify (such as changing to bilateral electrode placement), or discontinue ECT in nonresponders, as further improvement becomes less likely and the risk of side effects increases (17). However, limited research has evaluated outcomes after continuing treatment in patients who exhibited no response at a time point when the majority have usually responded to the treatment.

To address these clinically important questions, in the current study, we aimed to investigate the presence and characteristics of potential patient subclasses within symptom trajectories in ECT series, combining data-driven and more conventional statistical analyses. Additionally, we aimed to single out the subgroup with nonresponse to ECT in the vicinity of 9 treatments, evaluate their final outcomes, and further investigate variables associated with achieving a final response despite an initial nonresponse.

## Methods and Materials

### Setting, Sample, and Diagnosis

The current study is a register-based cohort study. Data were extracted from the Regional Register for Neurostimulation Treatment in Western Norway (19). The register is consent based; approximately 85% of the total patient population consented to inclusion in the register. The current study included patients with either unipolar (ICD-10 codes F32.1–F32.3, F33.1–F33.3) or bipolar (F31.3–F31.5) depressive episodes who were treated at the ECT unit at Haukeland University Hospital between June 2013 and December 2023. If patients had more than 1 ECT series in the register, only the first series was included. Further details concerning the register and the procedure for assessments, diagnosis, and registration of psychotropic medication have been described previously (5).

### Electroconvulsive Therapy

A Thymatron System IV (Somatics) was used for administering ECT, usually 3 times a week, although adjustments such as reducing frequency to 2 times a week could be made when clinically indicated, for example because of cognitive side effects. An ECT series was defined as consecutive treatments with no more than 7 days between each session, that is, patients with a treatment gap exceeding 7 days for reasons such as concurrent somatic incidents or self-selected temporary cessation of treatment were excluded (*n* = 30). Thiopental was the preferred anesthetic and was administered at an average dose of 3.39 (SD 1.00) mg/kg. When necessary, propofol (mean dose = 1.92 [0.84] mg/kg) or etomidate (mean dose = 0.19 [0.04] mg/kg) were alternatives. Succinylcholine was used as a muscle relaxant with a mean dose of 0.99 (0.19) mg/kg. Electric stimulus was typically applied approximately 90 seconds after the neuromuscular blockade. Right unilateral (RUL) electrode placement was predominantly used, initially with a pulse width of 0.5 ms. Initial stimulus energy was determined by an age-based method (20). Adjustments, such as increasing the electrical dose or switching from UL to bilateral (BL) electrode placement, were considered if seizures were insufficient or depressive symptoms did not improve satisfactorily. If cognitive side effects were observed (either through weekly Mini-Mental State Examination scoring, observations by staff, or patient-reported concerns during presession assessments), modifications were considered. These may consist of increasing the interval between sessions, reducing charge, changing electrode placement from BL to RUL, or using an ultrabrief pulse width of 0.25 ms. Treatment was generally concluded upon achieving remission, encountering unacceptable side effects, or when further improvement was deemed unlikely. This could be due to patients reaching a plateau in symptom reduction across several sessions despite treatment optimization and clinical judgment indicating limited benefit relative to potential risks, prior treatment experiences, or individual factors such as comorbidities or psychosocial challenges that made the aim of full remission less likely.

### Assessments/Outcome Measures

Symptoms of depression were assessed by the treating clinician using the Montgomery–Åsberg Depression Rating Scale (MADRS) (21). A MADRS score of ≤10 at the end of the ECT series was defined as remission (7), and a reduction in MADRS score pre-/post-ECT ≥ 50% was defined as response (7). Posttreatment MADRS scores were typically assessed after the final treatment session, but if unavailable, scores from up to 2 sessions prior were used (85 patients of the total sample of 344). End-of-treatment assessments occurred on average 2.3 (SD 4.6) days posttreatment. Diagnoses and patient characteristics were gathered from clinical charts and patient interviews. The presence of psychotic symptoms was based on ICD-10 diagnosis. Duration of depressive episode was based on patient self-report and was recorded in weeks. Psychotropic medication use was recorded at the first treatment session.

### Statistics

We used LCMM (10) to identify latent classes based on the trajectory of MADRS scores throughout an ECT series. The MADRS total score was the dependent variable, with treatment session number included as a categorical fixed variable and participant/patient ID included as a random intercept. Given the exploratory nature of this analysis, no other indicator variables/covariates were included. As this study was based on routine clinical practice, assessments were conducted at varying time points after different numbers of treatment sessions, although ECT was generally administered 3 times a week with assessments after every third session. Consequently, we used session numbers, rather than time points, as the fixed explanatory variable, relating MADRS scores to the session number when the assessment occurred. We deemed this approach more valid as it accounts for the therapeutic effects being driven primarily by the treatments rather than the passage of time. The LCMM was fitted starting with a 1-class model, with further classes being added successively. Criteria for model fit was a lower Bayesian information criterion (BIC) and sample size–adjusted BIC, providing class size >50 (22,23). Group differences between LCMM classes were analyzed using analysis of variance (ANOVA) or Kruskal-Wallis tests for continuous variables and χ^2^ tests for categorical variables. If normality was violated, variables were log- or square-root transformed. For unequal variances, Welch’s ANOVA was used, followed by a post hoc Games-Howell test; otherwise, Tukey’s test was applied. For χ^2^ tests with more than 2 LCMM classes, *z* tests with Bonferroni correction were used. Fisher’s exact test was applied when expected cell counts were low. The significance level was set at .05.

To investigate possible predictors of LCMM group affiliation, we conducted a multinomial regression analysis (24). The independent variables were sex; age; duration of the current depressive episode; psychotic features; bipolar disorder; baseline MADRS score; and having previously received ECT and an antidepressant, antipsychotic, or benzodiazepine/z-hypnotic at commencement of treatment. Selection of pretreatment independent variables was based on previous research (7,9), clinical experience, and availability in the registry.

To investigate final response status in the nonresponder group at session 9, a logistic regression model was performed (24). For patients still in treatment at session 9, response status was based on MADRS score change from baseline to session 9; if this score was unavailable, data from up to 2 sessions prior were used. The nonresponders at session 9 who continued to >9 treatment sessions were included in a logistic regression analysis to identify predictors of final response. Included independent variables were age, sex, psychotic features, and duration of depressive episode.

The LCMM [R LCMM package (10)] analysis was performed and figures were created using R (25). All other analyses were performed using SPSS Statistics version 29 (IBM Corp.) (26).

### Ethics

Included patients provided a written consent to the Regional Register for Neurostimulation Treatment in Western Norway, approved by the Norwegian Data Protection Authority (Approval No. 2012/5490). The study was approved by the Regional Committee for Medical and Health Research Ethics (Reference No. 2018/2541) (ClinicalTrials.gov ID NCT05388461).

## Results

### Description of LCMM Classes

**Figure 1:**
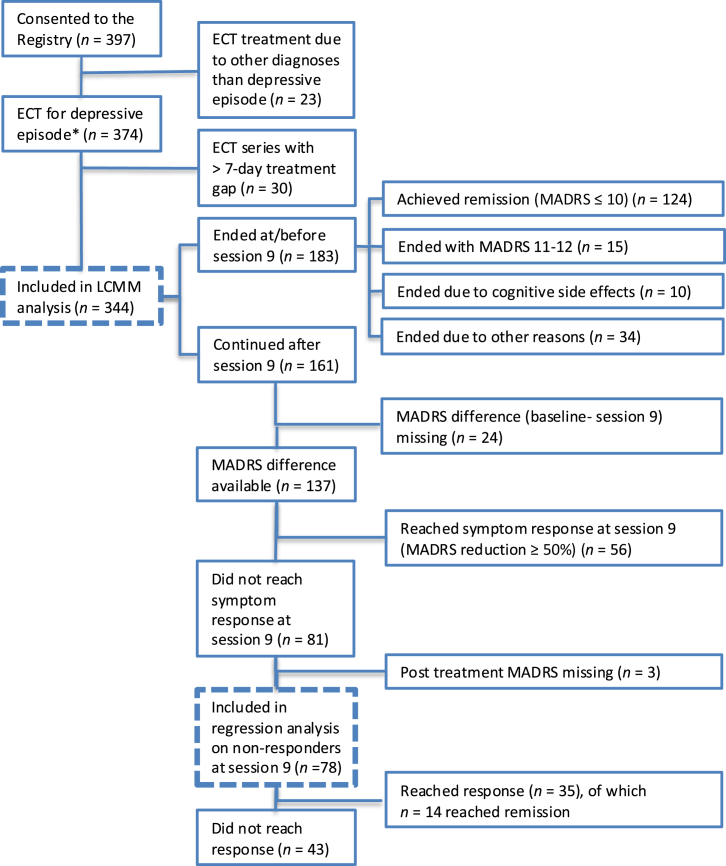
Flowchart of patient trajectories throughout electroconvulsive therapy (ECT) treatment. ∗ICD-10 codes F32.1–F32.3, F33.1–F33.3, and F31.3–F31.5. Dotted line indicates inclusion in main analyses. LCMM, latent class mixed model; MADRS, Montgomery–Åsberg Depression Rating Scale.

**Figure 2:**
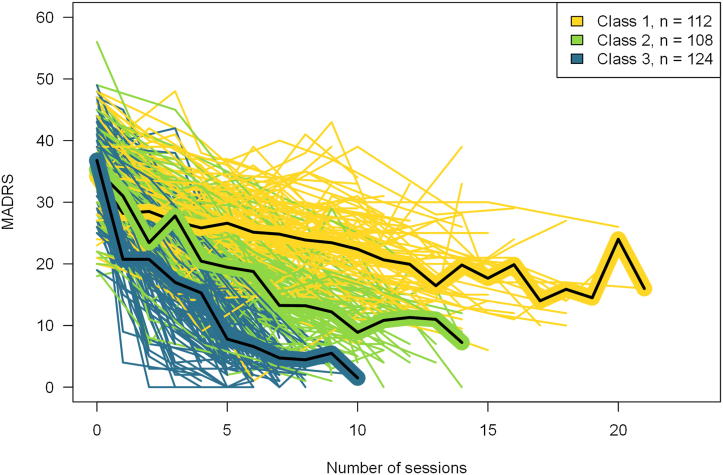
Latent class mixed model (LCMM) on Montgomery–Åsberg Depression Rating Scale (MADRS) score trajectories for 344 patients receiving electroconvulsive therapy. LCMM class 1, gradual improvement (*n* = 112); LCMM class 2, intermediate improvement (*n* = 108); LCMM class 3, fast improvement (*n* = 124).

The study included 344 patients (Figure 1); patient and treatment characteristics and LCMM group differences are shown in Table 1. The LCMM resulted in 3 classes; class 1 = gradual improvement (*n* = 112), class 2 = intermediate improvement (*n* = 108), and class 3 = fast improvement (*n* = 124). The model had an entropy value of 66.6, and the average posterior probabilities were 86.1% (class 1), 79% (class 2), and 87.3% (class 3). Robustness was assessed using multiple random starting values, yielding identical class solutions with proper convergence and a positive definite Hessian. Figure 2 shows MADRS score trajectories for each class over the course of the ECT treatments. Class 1 had a slower and more shallow improvement as compared with the other classes, with a mean baseline MADRS score of 34.2 and a mean posttreatment MADRS score of 20.9, after an average of 12.4 treatments. For class 2, the mean MADRS score at baseline was 35.3, the mean posttreatment MADRS score was 9.8, and the mean number of treatments was 10.3. Finally, in class 3, characterized by a rapid and steep trajectory, the mean baseline MADRS score was 36.8, the mean MADRS score posttreatment was 5.3, and the mean number of treatments was 6.3.

**Table 1:** Clinical and Demographical Characteristics of the Total Sample (*N* = 344) and LCMM Class Differences for Patients Receiving ECT for Depression

	Total Sample, *N* = 344	Class 1*, n* = 112	Class 2, *n* = 108	Class 3, *n* = 124	*p* Value	*p* Value, Bonferroni∗
Age, Years	53.8 (17.6)	46.8 (18.4)_a_	56.5 (17)_b_	57.7 (15.7)_b_	<.001^a^	<.001
Duration of Current Episode, Weeks	32.6 (60.2)	49.9 (97.6)_a_	28.2 (29.1)_a,b_	21.6 (26.5)_b_	.005	.085
Baseline MADRS Score	35.5 (6.3)	34.2 (5.9)_a_	35.3 (6.8)_a,b_	36.8 (6.0)_b_	.008	.136
MADRS Score After ECT	–	20.9 (7.4)	9.8 (4.9)	5.3 (4.1)	–	–
Sex						
Female	210 (61.0%)	56 (50.0%)_a_	66 (61.1%)_a,b_	88 (71.0%)_b_	.004	.068
Male	–	56 (50.0%)_a_	42 (38.9%)_a,b_	36 (29.0%)_b_		
Previously Received ECT						
Yes	62 (18.0%)	16 (14.3%)	21 (19.4%)	25 (20.2%)	.452	–
No	–	96 (85.7%)	87 (80.6%)	99 (79.8%)		
Family History of Severe Psychiatric Disorders^b^						
Yes	207 (64.5%)	73 (67.0%)	59 (57.8%)	75 (68.2%)	.233	–
No	–	36 (33.0%)	43 (42.2%)	35 (31.8%)		
Bipolar Disorder						
Yes	73 (21.2%)	29 (25.9%)	20 (18.5%)	24 (19.4%)	.334	–
No	–	83 (74.1%)	88 (81.5%)	100 (80.6%)		
Depression Severity^c^						
Moderate	53 (15.4%)	–	–	–	–	–
Severe without psychotic features	189 (54.9%)	–	–	–	–	–
Severe with psychotic features	102 (29.7%)	–	–	–	–	–
Psychosis						
Yes	–	15 (13.4%)_a_	36 (33.3%)_b_	51 (41.1%)_b_	<.001	<.001
No	–	97 (86.6%)_a_	72 (66.7%)_b_	73 (58.9%)_b_		
Medication at First ECT Session^d^						
Antidepressant						
Yes	274 (79.7%)	89 (79.5%)	87 (80.6%)	98 (79.0%)	.958	–
No	–	23 (20.5%)	21 (19.4%)	26 (21.0%)		
Antipsychotic						
Yes	245 (71.2%)	80 (71.4%)	72 (66.79%)	93 (75.0%)	.375	–
No	–	32 (28.6%)	36 (33.3%)	31 (25.0%)		
Benzodiazepine/z-drug						
Yes	84 (24.4%)	25 (22.3%)	25 (23.1%)	34 (27.4%)	.617	–
No	–	87 (77.7%)	83 (76.9%)	90 (72.6%)		
Anticonvulsant						
Yes	21 (6.1%)	9 (8.0%)	6 (5.6%)	6 (4.8%)	.568	–
No	–	103 (92.0%)	102 (94.4%)	118 (95.2%)		
Lithium						
Yes	22 (6.4%)	9 (8.0%)	7 (6.5%)	6 (4.8%)	.605	–
No	–	103 (92.0%)	101 (93.5%)	118 (95.2%)		
No psychotropic medication						
Yes	15 (4.4%)	3 (2.7%)	6 (5.6%)	6 (4.8%)	.549	–
No	–	109 (97.3%)	102 (94.4%)	118 (95.2%)		
Treatment Parameters						
Electrode placement					.002^e^	.039
Electrode placement UL	324 (94.2%)	101 (90.2%)_a_	102 (94.4%)_a,b_	121 (97.6%)_b_		
Electrode placement BL	5 (1.5%)	0 (0.0%)_a_	3 (2.8%)_a_	2 (1.6%)_a_		
Electrode placement switch	15 (4.4%)	11 (9.8%)_a_	3 (2.8%)_a,b_	1 (0.8%)_b_		
Number of ECT sessions	9.5 (3.6)	12.4 (3.5)	10.3 (2.1)	6.3 (1.7)		
Charge	291.1 (118)	260.8 (128.7)_a_	317.7 (124.5)_b_	295.4 (94.4)_a,b_	.003^a^	.049
Pulse width^f^	0.49 (0.07)	159.1 (0.5)_a_	185.8 (0.5)_b_	173.0 (0.5)_a,b_	.033^g^	.561

Values are presented as mean (SD) or *n* (%). Percentages reported are valid percent. Comparisons of classes are done as described in Methods and Materials. Different subscript letters (_a,b_) indicates groups with significant difference on a .05 level. For example, for age, there is a significant difference between classes 1 and 2 and classes 1 and 3, but not classes 2 and 3.

∗*p* Values were adjusted for multiple comparisons using Bonferroni correction (17 tests).

ANOVA, analysis of variance; BL, bilateral; LCMM, latent class mixed model; ECT, electroconvulsive therapy; MADRS, Montgomery–Åsberg Depression Rating Scale; UL, unilateral.

a Assumption of equal variances violated, Welch’s ANOVA reported, and Games-Howell post hoc test applied.

b History of first- or second-degree relatives with severe depression, bipolar disorder, or schizophrenia.

c Based on registered ICD-10 diagnosis, including both unipolar and bipolar depression.

d Administered on a regular basis.

e Due to low expected cell count, Fisher’s exact test is reported.

f Mean pulse width presented; distribution of patients by protocol type is included in the Supplement.

g Assumption of normality violated, nonparametric Kruskal-Wallis test performed, mean rank and median reported for test.

### Multinomial Regression for Predictors of LCMM Class Affiliation

A multinomial logistic regression was performed to investigate variables associated with LCMM class affiliation (Table 2). Male sex, lower age, longer duration of depressive episode, no psychotic features, and a lower baseline MADRS score increased the odds of affiliation with LCMM class 1 versus class 3. Male sex also meant higher odds of affiliation within class 2 versus class 3. Sensitivity analyses including age and treatment parameters electrode placement, mean pulse width, mean charge, and mean thiopental mg/kg were performed for all statistically significant findings one by one. All findings, apart from sex, remained statistically significant in these analyses (see the Supplement).

**Table 2:** Multinomial Regression Predicting Group Affiliation With Groups Defined by Latent Class Mixed Model Analysis

	Class 1, *n* = 94			Class 2, *n* = 100		
	Exp(*B*)	CI Exp(*B*)	*p*	Exp(*B*)	CI Exp(*B*)	*p*
Sex	3.66	1.89–7.1	<.001	1.97	1.07–3.61	.029
Age	0.95	0.93–0.97	<.001	0.99	0.97–1.01	.192
Duration of Current Episode, Weeks	1.01	1.0–1.02	.046	1.01	1.0–1.02	.314
Psychotic Features	0.43	0.19–0.97	.042	1.21	0.63–2.3	.569
Baseline MADRS Score	0.93	0.88–0.98	.007	0.96	0.91–1.01	.080
Previously Received ECT	1.67	0.69–4.06	.26	1.18	0.55–2.53	.677
Bipolar Disorder	1.08	0.48–2.44	.853	0.98	0.46–2.1	.956
Antidepressant^a^	1.85	0.75–4.55	.179	0.88	0.42–1.86	.735
Antipsychotic^a^	0.87	0.42–1.8	.706	0.62	0.32–1.19	.148
Benzo/z-hypnotic^a^	1.3	0.58–2.91	.521	1.11	0.57–2.18	.764

Reference group is class 3 (*n* = 106). Statistically significant model, χ^2^_20,__*n*__= 300_ = 76.3, *p* < .001.

ECT, electroconvulsive therapy; Exp, exponentiated; MADRS, Montgomery–Åsberg Depression Rating Scale.

a Administered on a regular basis.

### Identifying and Analyzing the Nonresponse Subgroup at Session 9: Outcomes and Predictors of Final Response

**Figure 3:**
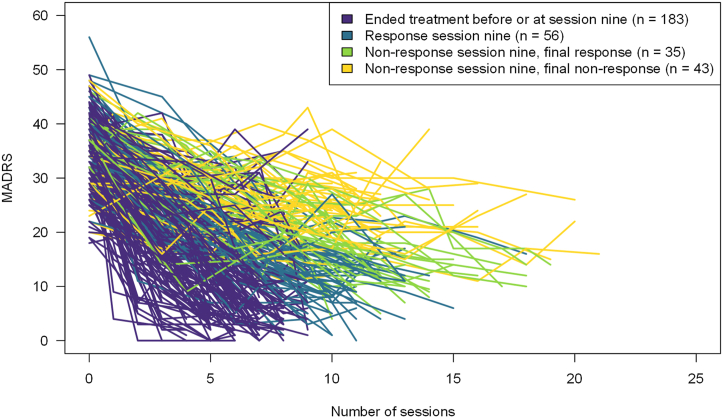
Montgomery–Åsberg Depression Rating Scale (MADRS) trajectories for individual patients receiving electroconvulsive therapy grouped by response status at treatment session 9 and final outcome.

A visual description of patient trajectories throughout the ECT series is shown in Figure 3, and a flowchart is shown in Figure 1. Of the 344 included patients, 183 (53%) ended their ECT series at or before session 9. Of these patients, 124 (67.8%) had achieved remission, defined as a MADRS score ≤ 10. Additionally, 15 (8.2%) patients ended treatment before session 9 with a MADRS score of 11 or 12, consistent with the remission criterion of a MADRS score ≤ 12 that was in place when the register was established. Finally, 10 (5.5%) patients ended treatment because of cognitive side effects, and 34 (18.6%) ended treatment because of unknown or other reasons, such as termination based on symptom improvement without remission, fear of future side effects, or somatic complications.

Among the 161 patients remaining in the ECT series at session 9, the MADRS difference score was available for 137 patients—that is, 24 (14.9%) patients were missing. Of the 137 patients, 56 (40.9%) patients had reached symptom response (MADRS reduction ≥50%) at this time while 81 (59.1%) had not. Three patients were missing a posttreatment MADRS score; accordingly, 78 patients were included in subsequent analyses. Of these 78 patients, 35 (44.9%) achieved response and 14 (17.9%) achieved remission (and response) at the end of treatment. The results of the regression analysis for achieving response at the end of treatment despite initial nonresponse are provided in Table 3. When including age as a continuous variable, the model was statistically significant, but Hosmer-Lemenshow test showed a somewhat poor fit. The relationship between age and ECT response was nonlinear, with 20% of responders in the 18 to 30 age group and 55.9% and 50% in the 31 to 60 and >60 age groups, respectively. As functional form may affect model fit (24), we transformed age into 3 categories (18–30, 31–60, and >60), resulting in the inclusion of an additional independent variable but improving model fit.

**Table 3:** Logistic Regression Predictors of Nonresponders at Treatment Nine Achieving Response After ECT

	Response^a^		
	Exp(*B*)	CI Exp(*B*)	*p*
Sex	3.04	1.00–9.23	.0497
Age 18–30, Reference			
Age 31–60	3.56	0.89–14.31	.073
Age >60	5.54	1.26–24.46	.024
Duration of Current Episode, Weeks	0.99	0.97–1.0	.103
Psychotic Features	1.68	0.35–8.19	.518

Statistically significant model, χ^2^_5,__*n*__= 70_ = 15.2, *p* = .009.

ECT, electroconvulsive therapy; Exp, exponentiated; MADRS, Montgomery–Åsberg Depression Rating Scale.

a MADRS score reduction ≥50% pre- to post-ECT.

Male patients and older patients (>60) were more likely to respond, with odds ratios of 3.04 (*p* = .0497) and 5.54 (*p* = .0238), respectively. In the group of patients with both an initial and final nonresponse, 3 patients (7.0%) had a worsening of depressive symptoms, 11 (25.6%) had a 1% to 25% improvement, and 29 (67.4%) had a 26% to 49% improvement.

## Discussion

We identified 3 trajectories of symptom improvement during ECT, highlighting heterogeneity in the pace and pattern of recovery despite overall benefit. While the fast (class 3) and intermediate (class 2) classes illustrate typical recovery, the gradual class (class 1) represents the greatest clinical challenge. In a multinomial regression analysis examining class affiliation, we found that a longer duration of the depressive episode, lower baseline MADRS score, younger age, and depression without psychotic features were associated with higher odds of affiliation to class 1 versus class 3.

Furthermore, among patients who did not respond to ECT by the first 9 treatment sessions, nearly one half (44.9%) eventually achieved response and 17.9% remission after continued treatment. Variables associated with final response in this subgroup were male sex and age >60.

By applying LCMM to a relatively large, real-world sample, including entire treatment series, our study adds new insight into the heterogeneity of ECT response trajectories, focusing on how symptom reduction evolves over time rather than only end-point outcomes. Previous studies have applied similar statistical methods to identify latent patient subgroups in the context of ECT (11, 12, 13), but findings have varied. For example, Cinar *et al.* (12) reported a no improvement class, and RheBergen *et al.* (13) found no link between psychotic features and class affiliation. These discrepancies may relate to limited sample sizes (<300) in previous studies, small latent class sizes, and methodological differences, including variations in the ECT technique. Notably, 2 of the studies mentioned above included a maximum of only 12 treatment sessions in their analyses.

While the identification of distinct response trajectories provides an understanding of how improvement unfolds during ECT, it is also important to consider how these patterns align with established predictors of treatment outcome (response or remission). Previous studies have linked older age and psychotic features to higher odds of response or remission (7), whereas longer episode duration has predicted poorer outcomes (9). The potential interdependence of such clinical predictors has also been highlighted in recent work. Jelovac *et al.* (27) performed a causal mediation analysis and found that 43.1% of the effect of older age on ECT response was explained by mediators including psychotic features, episode duration, and psychomotor disturbances. A prediction model from the Dutch ECT consortium (28) identified higher age, shorter duration of episode, and depression with psychotic features as indicators of better post-ECT outcomes for a lower depression score after ECT. In our analysis, incorporating several known predictors in multivariate models, we found similar associations, supporting the idea that age, episode duration, psychotic features, and baseline severity are relevant not only for final outcomes but also for understanding ECT symptom trajectories. This provides the context for the clinical considerations that follow. When recommending a specific treatment, clinicians must consider not only safety but also the likelihood of a favorable treatment outcome for the individual patient. Although pretreatment predictors of ECT response are becoming increasingly well documented, some of these factors reflect a generally poor prognosis across treatment options and are not unique to ECT. For example, a longer duration of depressive episode is a general prognostic marker of poorer treatment response across various modalities, including pharmacotherapy and psychotherapy (29), making treatment choice less straightforward. In contrast, certain predictors, such as older age and the presence of psychotic features, appear to be more specifically associated with favorable outcomes following ECT and may support recommending ECT over other treatment options. Our results show that not only end-point outcomes but also patient trajectories vary, and as more targeted forms of psychiatric therapies are developed, other treatment strategies may be relevant to consider. Pretreatment patient characteristics indicating lower odds of a favorable ECT outcome, combined with a shallow symptom improvement trajectory, may warrant evaluation of the treatment. This could include evaluating the impact of comorbidities, intensifying or augmenting the ECT treatment, or considering other treatment options, especially if there are cognitive side effects of ECT. Relevant to our study, ketamine has been suggested as a treatment alternative to ECT, particularly in younger patients (30). Repetitive transcranial magnetic stimulation has also been found to be effective in younger patients (31), and novel, more efficient treatment protocols are emerging (32). However, comparative evidence remains limited, and robust head-to-head trials are needed before such strategies can be routinely recommended. Clinical decisions remain complex (33) and must remain individualized, while also considering urgent indications such as suicidality, poor oral intake, and the overall mental state of the patient.

While pretreatment predictors can guide the initial choice of ECT, an equally important clinical challenge arises during the treatment course: deciding whether to continue or discontinue when early improvement is limited. Although Norwegian guidelines (34) mandate ongoing evaluation, they lack specific criteria for continuation or discontinuation. Husain *et al.* (35) examined whether early symptom thresholds could guide decisions to continue or discontinue treatment. They found that patients achieving at least a 30% reduction in depressive symptoms by session 7 were more likely to respond later, whereas patients below this threshold had a lower chance of improvement. Our results suggest that early nonresponse does not necessarily indicate futility and should not automatically lead to treatment termination, as a substantial proportion of patients may still achieve meaningful improvement later in the course. Communicating this possibility to patients can help maintain hope and adherence. These findings emphasize that early symptom change is informative, but not definitive, for treatment decisions.

In our study, we also conducted a regression analysis to understand the factors influencing the final response in patients who did not respond to treatment initially. Our data suggest that younger female patients have lower odds of achieving a final response. As the sample size is limited, which limited the number of independent variables that we were able to include, we urge that these results be interpreted with caution. Previous research has not found sex differences in ECT outcome (36). However, it is plausible that a patient group not achieving response to ECT at a time point when most patients have responded or even remitted represents a subgroup with different clinical features and treatment responses. In addition, it is noteworthy that a previous study based on a large Swedish sample found that younger female patients were more likely to report subjective memory worsening following ECT (37). In conclusion, larger, better-powered studies that include additional relevant variables, such as comorbidities, are needed.

### Strengths and Limitations

A strength of this study was the large sample with regular assessments during the entire course of treatment. Our sample reflects a broad clinical population, which strengthens the ecological validity of our findings (38). However, as this is a consent-based register, there is a risk of selection bias as data are unavailable for nonconsenting patients. In a registry study, factors such as missing data, variation in time points for assessments, and no standardized or uniform procedure for diagnosis also represent possible limitations (38). Assessments were also not necessarily done by the same clinicians during treatment; these could be performed, for example, by trained nurses, junior doctors, or experienced psychiatrists. Although raters are trained clinicians, and assessments followed standard procedures, we cannot rule out the possibility of this biasing estimates. In some patients, treatment was changed during the ECT series (e.g., reduced frequency to ≤2 sessions/week or other adjustments of treatment parameters), typically in response to clinical or cognitive considerations, which might have affected trajectory estimates. The sample of patients showing nonresponse at treatment 9 was small, limiting the inclusion of independent variables in the regression model (39). Information on medication resistance or comorbidities is not available in the registry. These are factors that may affect outcome after ECT (40,41), and not being able to include them represents a limitation (42).

### Conclusions

Our study sheds further light on the presence of diverging response patterns of symptom alleviation through ECT treatment series, identifying a patient subclass with a less favorable symptom improvement trajectory in routine care and factors associated with affiliation to this class.

The decision to continue or discontinue ECT in patients showing an initially nonresponsive trajectory remains clinically challenging. Our findings emphasize the importance of individualized, response-guided decisions about whether to continue, optimize, or taper ECT, balancing expected benefit with side effects and patient priorities.

## Supplementary Material

Tables S1 and S2

Key Resources Table
