# Supplementary material for: Diverging Trajectories of Depressive Symptoms During Electroconvulsive Therapy: Toward Personalized Treatment
Source: Biol Psychiatry Glob Open Sci. 2026 Mar 13;6(4):100722. doi: 10.1016/j.bpsgos.2026.100722 (PMC13141716; doi:10.1016/j.bpsgos.2026.100722)
Supplement: Tables S1 and S2 [file mmc1.pdf]

SUPPLEMENTARY INFORMATION

Diverging Trajectories of Depressive Symptoms During Electroconvulsive Therapy: Towards Personalized Treatment

Sellevåg *et al.*

Table S1

Sensitivity analyses including treatment parameters and statistically significant variables from primary analysis, investigated one-by-one. Primary analysis identified latent classes of patient trajectories of depressive symptoms throughout ECT series and variables associated with LCMM class affiliation.

	Class 1		Class 2	
	Exp.(B)	p	Exp.(B)	p
Sex	0.77	0.453	1.27	0.487
Age	0.92	<0.001	0.95	0.013
Duration current episode[a]	1.01	0.025	1.01	0.194
Psychotic features	4.19	<0.001	1.39	0.261
Baseline MADRS	0.92	<0.001	0.96	0.064

Note. Reference group: Class 3.
[a]Duration in weeks
ECT = Electroconvulsive therapy; LCMM: Latent Class Mixed Model; MADRS: Montgomery-Åsberg Depression Rating Scale

Table S2

Distribution of mean Pulse Width in Sample of 344 patients receiving ECT for depression

	n (%)
0.25 ms	7 (2)
0.26-0.49 ms*	49 (14.2)
0.5 ms	258 (75)
> 0.5 ms**	30 (8.7)

Note.
*Patients initiating treatment series with pulse width of 0.5 ms but reducing it during series due to subjective cognitive side-effects.
**The Thymatron system will automatically adjust pulse width to > 0.5 ms when charge exceeds 504 mC, resulting in mean pulse width > 0,5 ms.
ECT = Electroconvulsive therapy
